# Supplementary material for: Nanofiber-interwoven gel membranes with tunable 3D-interconnected transport channels for efficient CO2 separation
Source: Nat Commun. 2025 Sep 2;16:8199. doi: 10.1038/s41467-025-63502-2 (PMC12405441; doi:10.1038/s41467-025-63502-2)
Supplement: Supplementary file 1 — Supplementary Information [file 41467_2025_63502_MOESM1_ESM.pdf]

Supplementary Information for

Title: Nanofiber-interwoven gel membranes with tunable 3D-interconnected transport channels for efficient CO_2 separation

Authors: Hao-Nan Li,[1,2]† Ze-Yu Sun,[1,2]† Zhen-Jie Yu,[1,2] Ke-Xin Man,[1,2] Chao Zhang,[1,2]* Zhi-Kang Xu[1,2,3]*

Affiliations:

[1]MOE Key Laboratory of Macromolecular Synthesis and Functionalization, and Key Laboratory of Adsorption and Separation Materials & Technologies of Zhejiang Province, Department of Polymer Science and Engineering,
Zhejiang University,
Hangzhou, 310058, China
[2]The "Belt and Road" Sino-Portugal Joint Lab on Advanced Materials, International Research Center for X Polymers,
Zhejiang University,
Hangzhou, 310058, China
[3]Institute of Marine Chemistry and Environment, Ocean College,
Zhejiang University,
Zheda Road 1, Zhoushan 316000, China
*Corresponding author. Email: zhangchao7@zju.edu.cn (C. Zhang), xuzk@zju.edu.cn (Z.-K. Xu)
† These authors contributed equally to this work

This file includes:

Supplementary Methods

Supplementary Figures 1 to 25

Supplementary Tables 1 to 3

Supplementary References

Supplementary Methods

Molecular dynamics simulation

CNT molecule model with inner diameter of 8 Å was constructed by UFF force field. PEG molecule chain model was constructed by 20 repeat units each chain and parametrized by the GAFF force field. The restrained electrostatic potential (RESP) charges were fitted after using density functional theory (DFT) for geometry optimization and single point energy calculation based on B3LYP functional with D3BJ dispersion correction at the B3LYP/TZVP level. The pure GMs constructed by 54 PEG chains and the NIGMs consisting of 16 CNT molecules and 50 PEG chains were respectively added in a cubic simulation box and performed 21 steps molecular dynamic compression and relaxation[1]. It was worth noted that the CNT molecule in composite model was oriented perpendicular to z axis and applied a position restriction with a force constant of 10 $kJ \cdot mol^{-1} \cdot nm^{-2}$ along z direction to avoid random distribution. The free volumes of pure GMs and NIGMs after 21 steps were counted by adopting grid interval of 0.25 Å and connolly probe radius of 1.0 Å.

CO_2 diffusion simulation of membranes: All molecular dynamic simulations were executed by using GROMACS 2023 software package. CO_2 molecules parametrized by the GAFF force field with the RESP charges were randomly packed into composite membrane system. Before performing NPT simulation, the system was executed a process of energy minimization by steepest descent method with the force tolerance of 10 $kJ \cdot mol^{-1} \cdot nm^{-1}$ and executed a process pre-equilibrium simulation of 20 ns NPT process. Subsequently, mean square displacements (MSD) of CO_2 nearby PEG matrix and nearby CNT molecules were respectively counted during a 100 ns equilibrium NPT simulation to eventually obtain their diffusion coefficients (D) according to following equation (1): $\mathrm{D} = \lim_{t \to \infty} \frac{1}{6t} \left(\frac{1}{N} \sum_{N}^{k=1} |r_k(t) - r_k(0)|^2 \right)$ (1)

where $r_k(t)$ is the position of the *k*th molecule at time *t* and *N* is the number of molecules.

Supplementary Figures

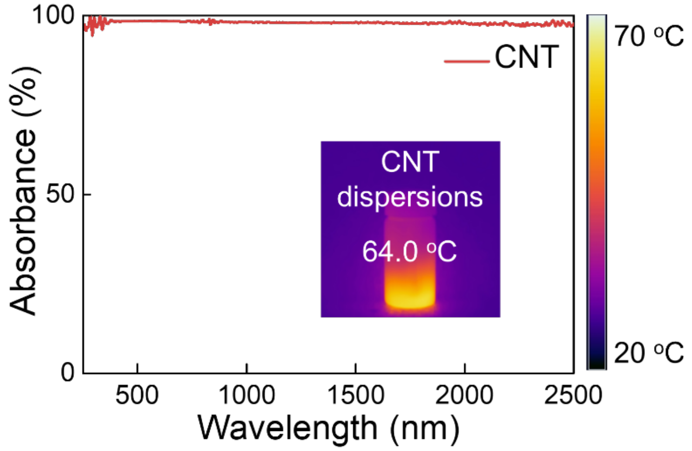

Supplementary Fig. 1 Absorption spectra of CNT-interwoven photothermal confined reactor. Insets show the photothermal-induced temperature varying of CNT dispersions.

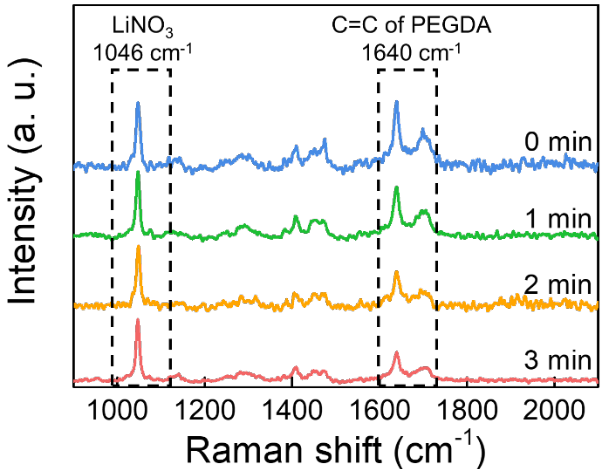

Supplementary Fig. 2 Raman spectra of NIGMs under different photothermal-triggered gelation times.

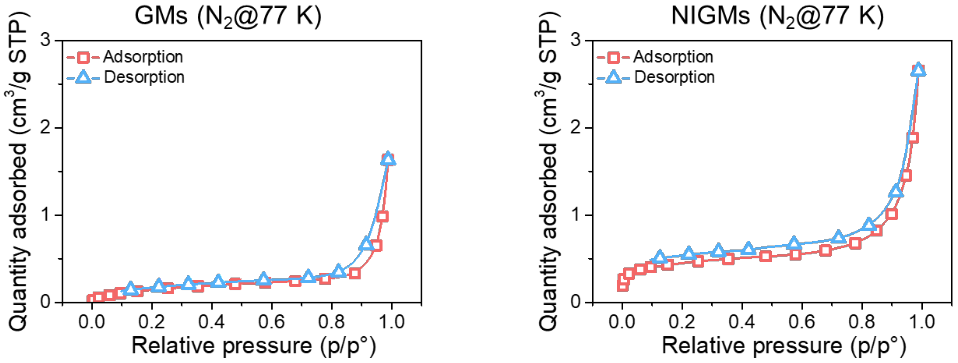

Supplementary Fig. 3 Nitrogen adsorption-desorption isotherms of pure GMs and NIGMs at 77 K.

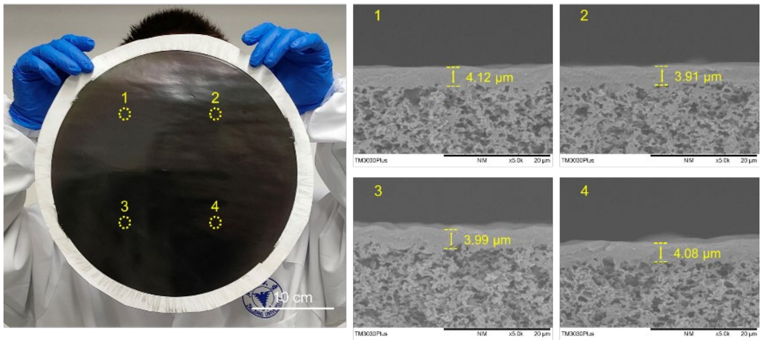

Supplementary Fig. 4 30-cm-diameter NIGMs with uniform thickness.

The 30-cm-diameter NIGMs were fabricated as follows: Initially, a 30 cm-diameter CNT-interwoven photothermal reactor was fabricated using a customized large vacuum filtration device (Supplementary Fig. 5a). Subsequently, the CNT-interwoven reactor was uniformly sprayed with gel precursors (0.13 g·cm^{-2}) by spray gun. The precursor-infused photothermal reactor was then exposed to a multi-Xenon lamp system (Supplementary Fig. 5b), enabling large-area fabrication of NIGMs through photothermal-triggered in-situ gelation method. Moreover, we also proposed a viable route to further scale up the photothermal-triggered fabrication method by coupling vacuum filtration with spraying, as illustrated in Supplementary Fig. 6. To make sunlight-based gelation feasible for industrial-scale membrane production, we employed a multi-Xenon lamp array system to expand the projected solar irradiation area, thus paving the way for industrial-scale NIGMs production via our photothermal-triggered gelation method.

In addition to the sunlight-based gelation, we also investigated the photothermal effects of the CNT-interwoven confined reactor under irradiation of multi-UV light system (Supplementary Fig. 7). Given that the CNT-interwoven reactor exhibits a 98.6% absorption across the ultraviolet spectrum (Supplementary Fig. 1), its surface temperature rapidly escalates to 78.6 °C within 120 seconds under UV exposure (1500 W·m^{-2}). This rapid photothermal response of CNT-interwoven reactor provides a thermal basis for initiating in-situ gelation of precursors, demonstrating the feasibility of employing multi-UV light systems for scalable NIGMs production via our photothermal-triggered in-situ gelation method.

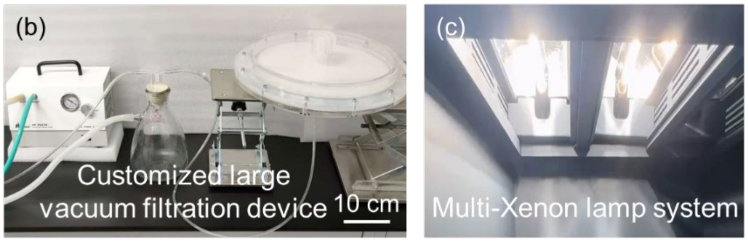

Supplementary Fig. 5 Digital images of (a) customized large vacuum filtration device and (b) multi-Xenon lamp system.

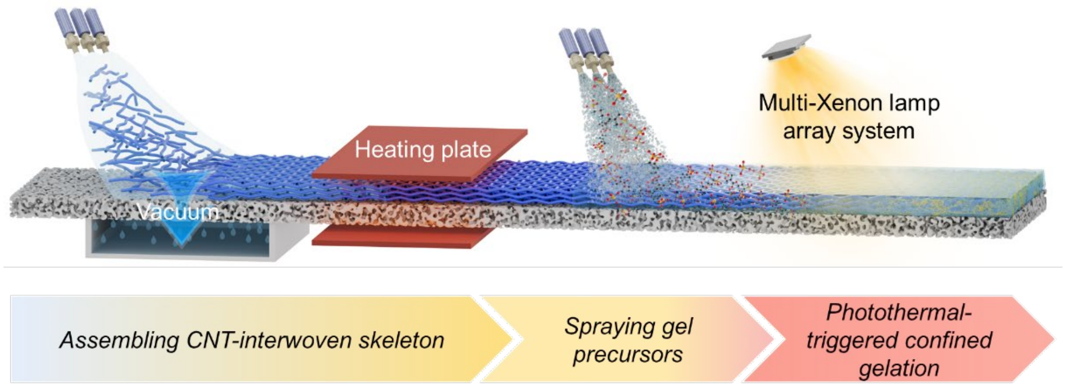

Supplementary Fig. 6 Schematic diagram of the industrial-scale fabrication of NIGMs through photothermal-triggered in-situ gelation method.

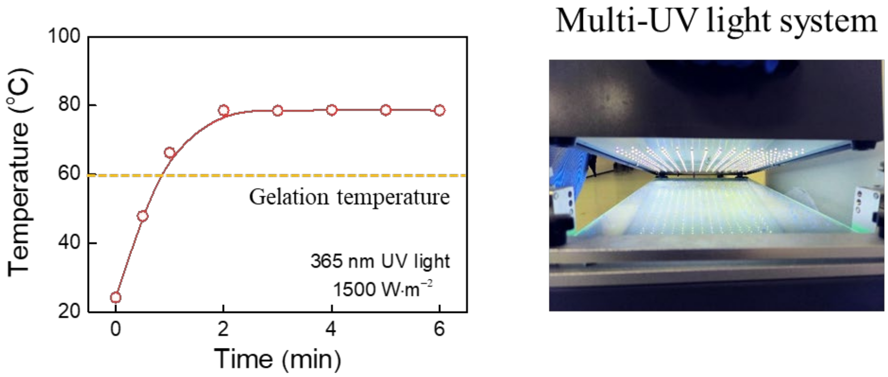

Supplementary Fig. 7 Dynamic curve of temperature of the CNT-interwoven photothermal confined reactor under irradiation of multi-UV light system.

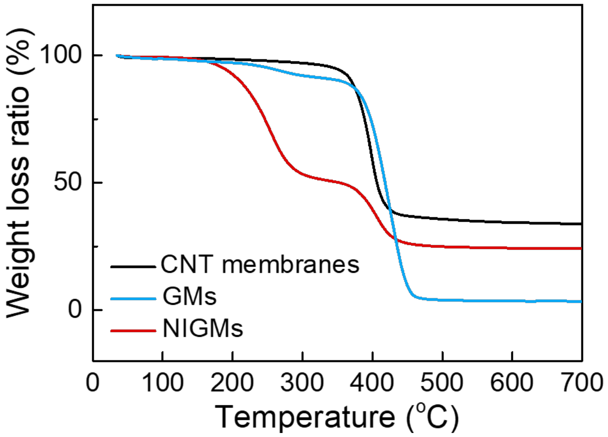

Supplementary Fig. 8 Thermo-gravimetric curves of CNT membranes, GMs and NIGMs in nitrogen. The resulting NIGMs shows an ultrahigh nanomaterial loading contain a CNT content of up to 66.7%. TGA in nitrogen gas can be employed to monitor the degree of CNT functionalisation. In the case of CNT membranes, a 66.4 wt % loss at 700 °C relative to the pristine material is observed, corresponding to 15.5 mmol COOH/g CNT.

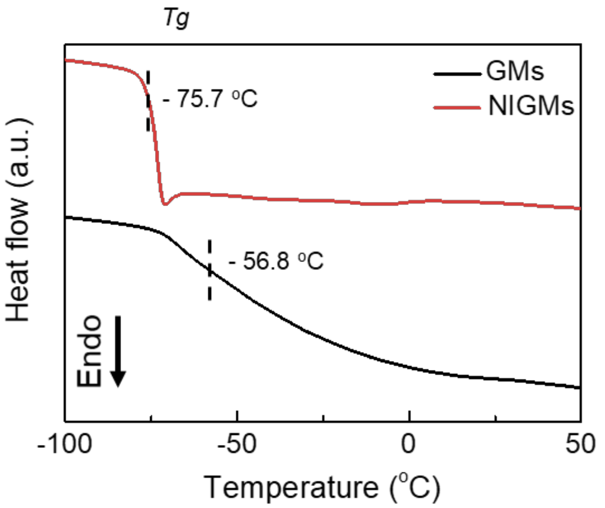

Supplementary Fig. 9 DSC curve of gel and NIGMs. The glass transition temperature decreases from -56.8 °C in the GMs to -75.7 °C in the NIGMs. This reduction underscores the heightened mobility of polymer chains within NIGMs.

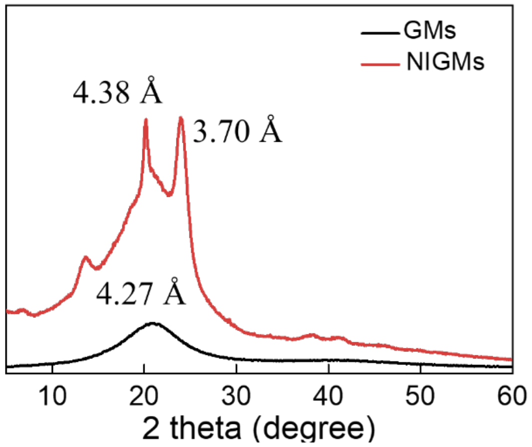

Supplementary Fig. 10 XRD patterns and average chain *d*-spacing of GMs and NIGMs. The CNT-interwoven skeleton of NIGMs disrupts the stacking of polymer chains within gel networks for leading to increased free volume for facilitating CO_2 diffusion and permeance.

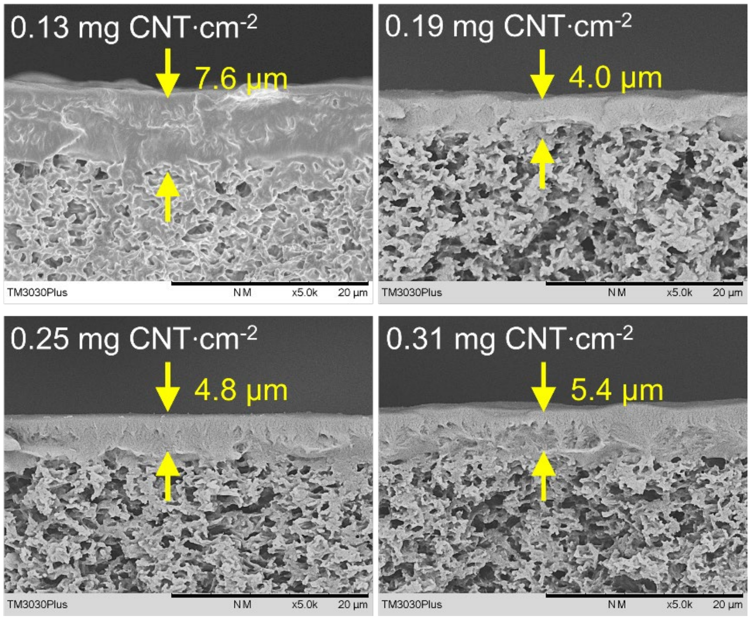

Supplementary Fig. 11 Cross-sectional morphologies of the NIGMs with different stacking densities of CNT-interwoven skeleton. When the CNT packing density is insufficient (< 0.19 mg·cm^{-2}), the gel precursor is prone to penetrate into the underneath porous membrane, subsequently yielding a pure gel layer underneath the CNT-interwoven skeleton during photothermal-triggered gelation process.

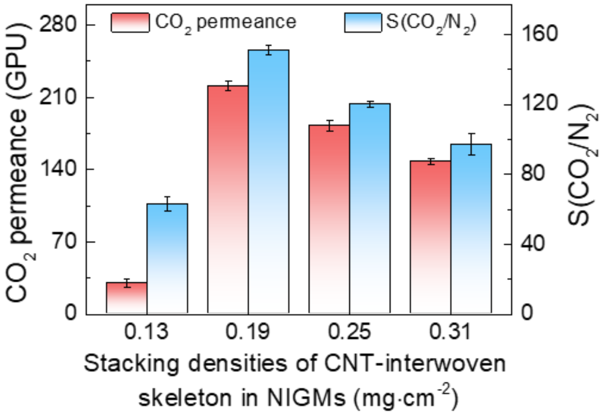

Supplementary Fig. 12 CO_2 separation performance of the NIGMs with different stacking densities of CNT-interwoven skeleton. Data presented as mean ± SD, n = 5.

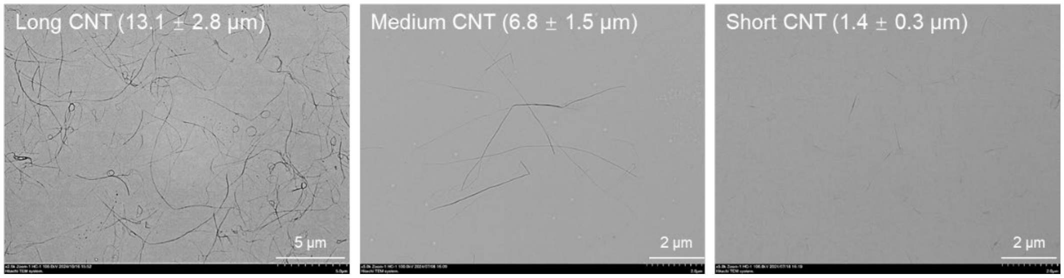

Supplementary Fig. 13 TEM images of CNT with different average lengths from 13.1 ± 2.8 μm (long CNT) to 6.8 ± 1.5 μm (medium CNT) and 1.4 ± 0.3 μm (short CNT).

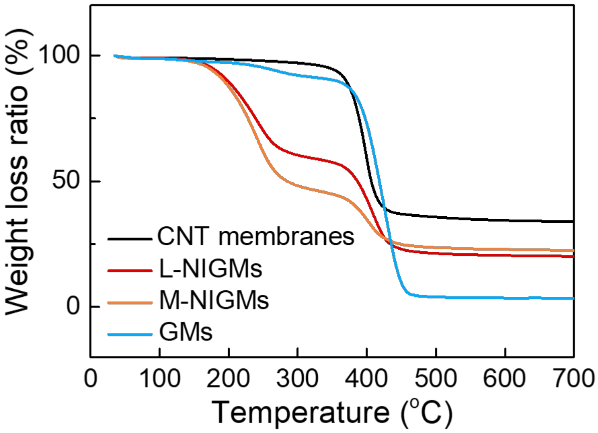

Supplementary Fig. 14 Thermo-gravimetric curves of CNT membranes, GMs, L-NIGMs and M-NIGMs in nitrogen. The CNT loadings of L-NIGMs and M-NIGMs are 60.3% and 64.7%.

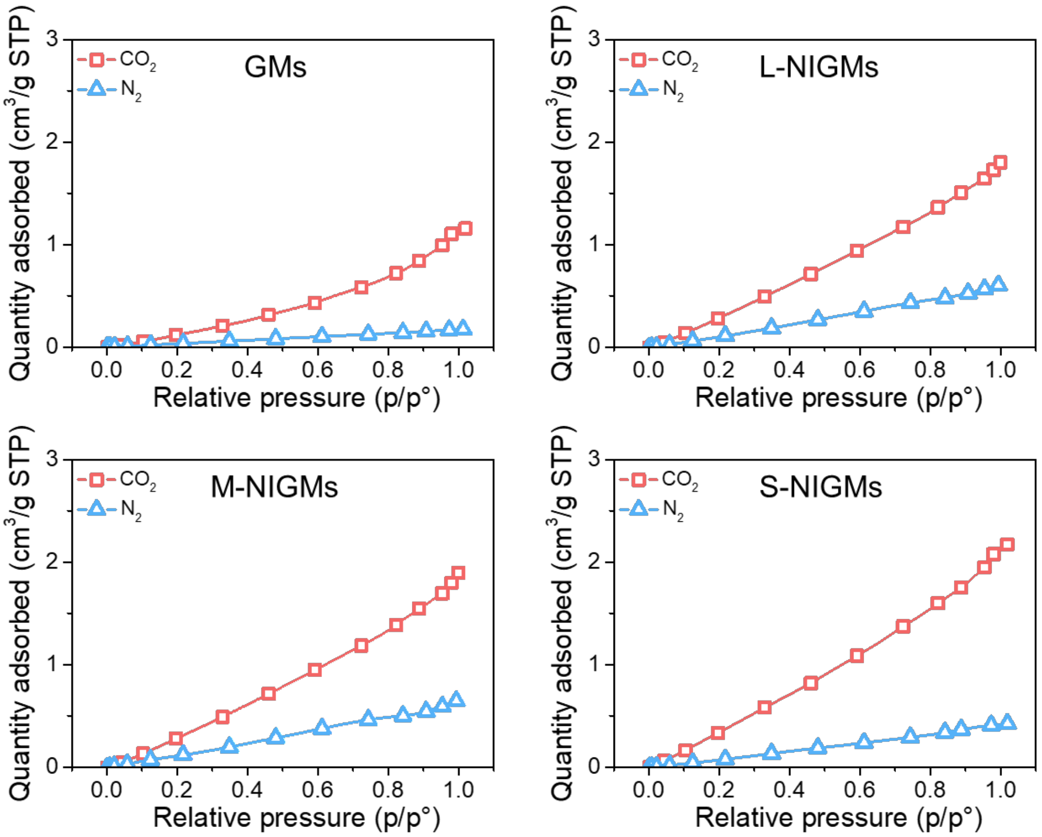

Supplementary Fig. 15 CO_2 and N_2 adsorption isotherm of GMs and various NIGMs at 298 K.

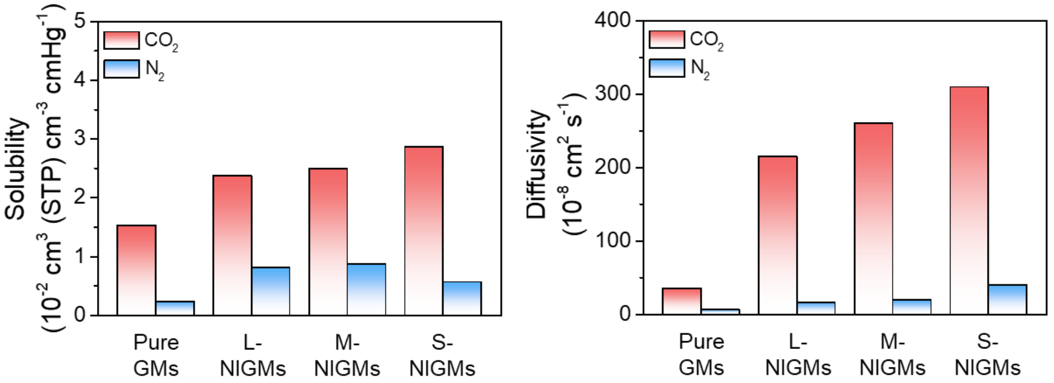

Supplementary Fig. 16 Solubility (left) and diffusivity (right) variation of CO_2 and N_2 for GMs and various NIGMs.

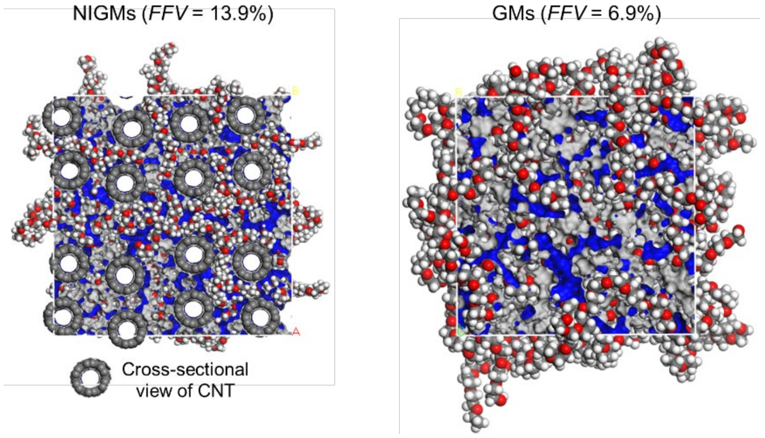

Supplementary Fig. 17 Fractional free volume for the NIGMs and pure GMs via molecular dynamics simulation.

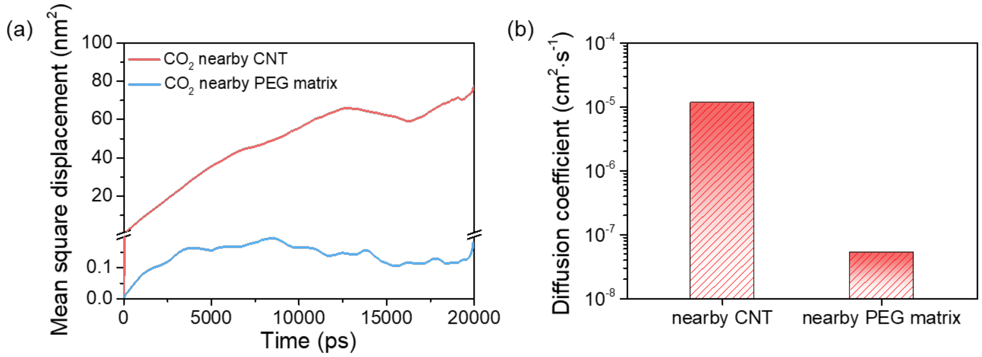

Supplementary Fig. 18 (a) Mean square displacement and (b) diffusion coefficients of CO_2 nearby PEG matrix and nearby CNT within NIGMs.

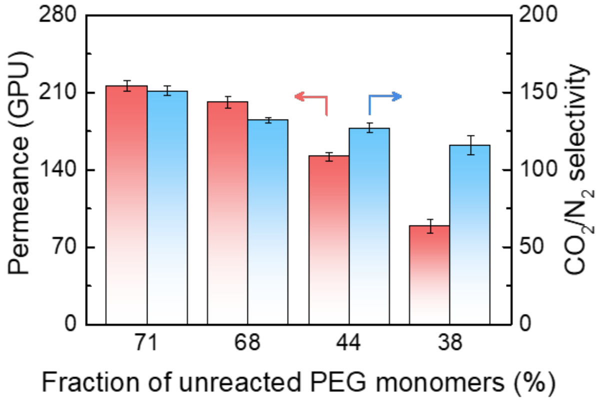

Supplementary Fig. 19 CO_2 permeance and CO_2/N_2 selectivity of various NIGMs with different fractions of unreacted PEG monomers. By controlling the conversion of PEG monomer, the NIGMs can accommodate some unreacted PEG monomer as function additive to enhance the mobility of polymer chains as well as act as CO_2 carriers within gel network, fostering a more facile diffusion of CO_2. Data presented as mean ± SD, n = 5.

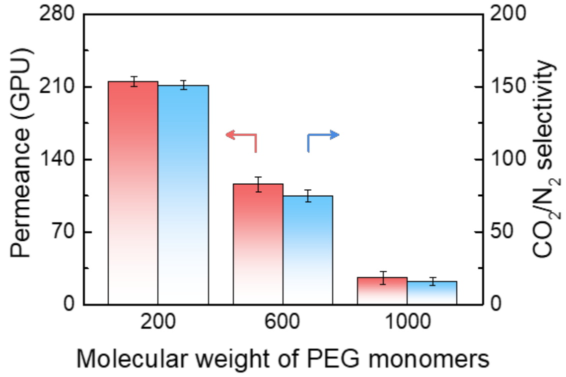

Supplementary Fig. 20 CO_2 permeance and CO_2/N_2 selectivity of various NIGMs with different molecular weight of PEG monomers. The polymer chains of NIGMs fabricated by higher molecular weight of PEG monomer are prone to form intramolecular and intermolecular interactions and even occur local crystallization, which impedes CO_2 diffusion and consequently reduces CO_2 permeance. Data presented as mean ± SD, n = 5.

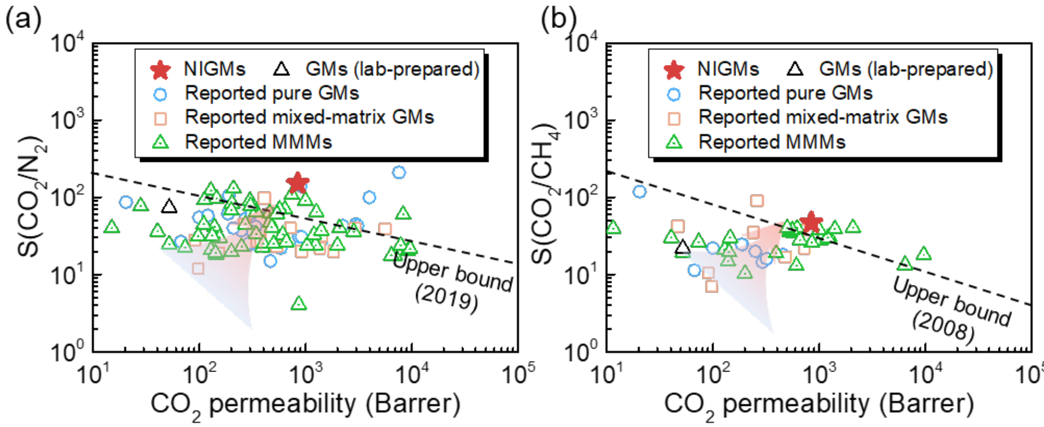

Supplementary Fig. 21 (a) Comparative evaluation of NIGMs against reported GMs and reported MMMs in terms of CO_2 permeability and CO_2/N_2 selectivity. (b) Comparative

evaluation of NIGMs against reported GMs and reported MMMs of CO_2 permeability and CO_2/CH_4 selectivity.

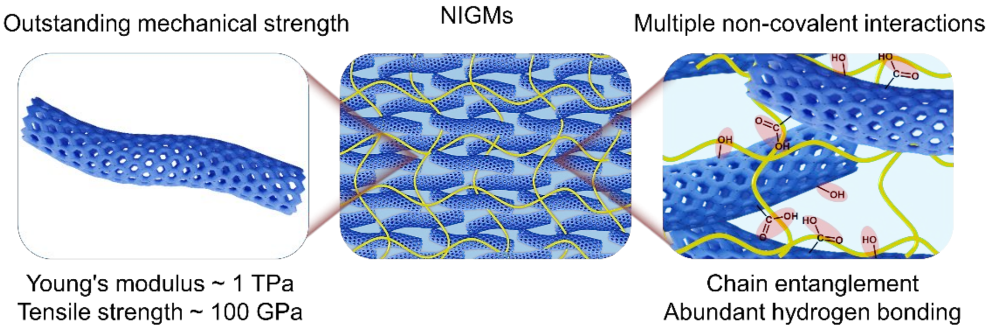

Supplementary Fig. 22 Schematic representation of the excellent structural stability of NIGMs.

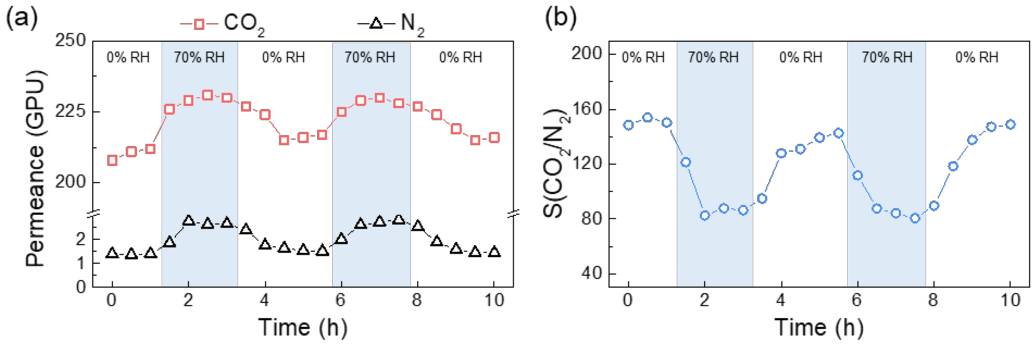

Supplementary Fig. 23 (a) CO_2 permeance and (b) selectivity of NIGMs varied humidity cycling.

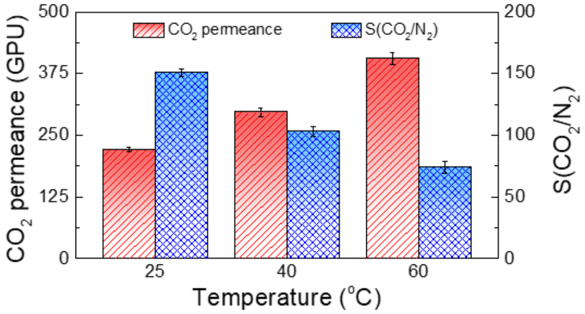

Supplementary Fig. 24 CO_2 separation performance of NIGMs under different temperatures. Data presented as mean ± SD, n = 5.

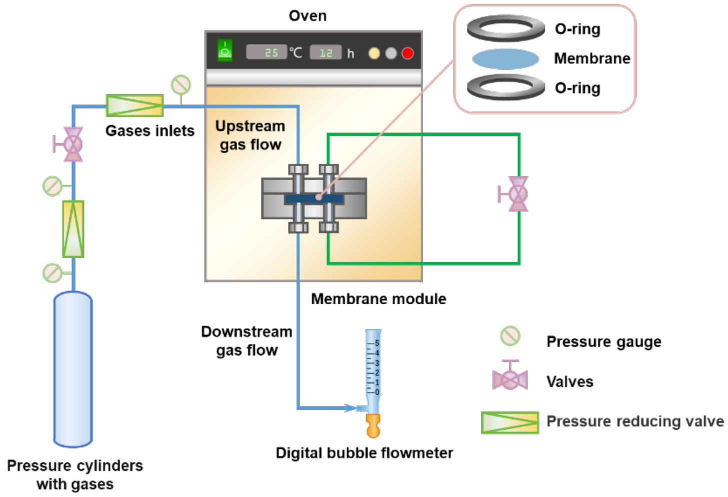

Supplementary Fig. 25 Schematic diagram of pipeline engineering for gas permeation test

Supplementary Tables

Supplementary Table 1 Comparison of the NIGMs and reported MMMs in terms of the improvement of CO_2 permeability and CO_2/N_2 selectivity.

Membrane type	CO_2 permeability variation (%)	CO_2/N_2 selectivity improvement (%)	References
MOF-based MMMs	200	-8.3	2
MOF-based MMMs	3.1	5.2	3
MOF-based MMMs	30.7	64.3	4
MOF-based MMMs	242.8	0	5
MOF-based MMMs	958	-44.4	6
MOF-based MMMs	141.6	62.5	7
MOF-based MMMs	1180	5.7	8
MOF-based MMMs	88.9	25	9
MOF-based MMMs	66.7	31.7	10
MOF-based MMMs	99.4	50	11
MOF-based MMMs	296.1	89.6	12
MOF-based MMMs	102.7	65.9	13
MOF-based MMMs	52.4	44.3	14
MOF-based MMMs	165	32.3	15
MOF-based MMMs	1.4	100	16
MOF-based MMMs	67	33	17

MOF-based MMMs	85.6	88.7	18
COF-based MMMs	52	32	19
COF-based MMMs	70.4	18.8	20
COF-based MMMs	120	111	21
COF-based MMMs	112.8	26.4	22
COF-based MMMs	107.4	-15.3	23
Zeolite-based MMMs	85.9	26.3	24
Zeolite-based MMMs	44.9	117.9	25
Zeolite-based MMMs	77.7	100	26
POP-based MMMs	140	22	27
POP-based MMMs	649	-7.3	28
POP-based MMMs	151.2	-1.2	28
POP-based MMMs	283.4	-3.4	28
POP-based MMMs	154.2	2.2	28
POP-based MMMs	177.3	-0.2	28
POP-based MMMs	195.4	-15.4	29
POP-based MMMs	58.8	-24.2	30
POP-based MMMs	223.5	-3.1	30
Nanosheet-based MMMs	15.7	300	31
Nanosheet-based MMMs	101.4	29.4	32

Nanosheet-based MMMs	102.3	42.2	33
Nanosheet-based MMMs	100.3	55.5	34
Nanosheet-based MMMs	925	37.5	35
Nanosheet-based MMMs	405.5	80.5	35
Nanosheet-based MMMs	250	127.6	37
Nanofiber-based MMMs	106	0	38
Nanofiber-based MMMs	290.1	51.8	39
Nanofiber-based MMMs	86.7	27.3	40
Nanofiber-based MMMs	90	13	41
Nanofiber-based MMMs	25	13.3	42
Nanofiber-based MMMs	230	270	43
**NIGMs**	**1558**	**287**	**This work**

Supplementary Table 2 Comparison of the NIGMs and reported GMs and MMMs in terms of the CO_2/N_2 separation performances.

Year	Membrane type	CO_2 permeance (GPU)	CO_2 permeability (Barrer)	CO_2/N_2 selectivity	References
2012	pure GMs	2.3	340	42	44
2012	pure GMs	1.8	273	53	44
2013	pure GMs	51	910	25	45
2014	pure GMs	6100	585.6	22	46
2015	pure GMs	6.7	4000	100	47
2015	pure GMs	20	900	140	48
2017	pure GMs	11.9	2980	45	49
2017	pure GMs	4.7	190	105.6	50
2017	pure GMs	95	7600	210	51
2018	pure GMs	1.25	100	55	52
2018	pure GMs	1.7	340	31	53
2018	pure GMs	0.6	120	58	53
2018	pure GMs	1.4	290	24	53
2018	pure GMs	1.1	210	40	53
2020	pure GMs	0.2	20.4	86.8	54
2020	pure GMs	2.9	292	25.4	55
2021	pure GMs	1.3	66.9	26.7	56
2022	pure GMs	3.5	319.1	35.9	57
2022	pure GMs	3.5	1421	27	58
2022	pure GMs	778	466.8	15	59
2022	pure GMs	6.5	456.4	61.4	60
2023	pure GMs	5.6	2240	43	61
2024	pure GMs	2.0	186.4	61.7	62
2024	pure GMs	10.3	3100	43	63
2024	pure GMs	2~10	500~2920	61	64
2024	pure GMs	4.3	866	31	65
2024	pure GMs	2.3	464	63	65
2025	pure GMs	98.2	913.3	31	66
2025	pure GMs	3.1	310	62	67
2017	Mixed-Matrix GMs	19.3	2910	40	68
2017	Mixed-Matrix GMs	3.1	153.6	19.1	69
2017	Mixed-Matrix	9.2	1850	20	70

	GMs				
2019	Mixed-Matrix GMs	350	350	27	71
2019	Mixed-Matrix GMs	144	1440	31.2	72
2021	Mixed-Matrix GMs	3.1	920	20	73
2021	Mixed-Matrix GMs	119	535	23	74
2021	Mixed-Matrix GMs	0.8	98	12	75
2021	Mixed-Matrix GMs	4.5	1362	22	76
2022	Mixed-Matrix GMs	0.7	90.6	27.8	77
2023	Mixed-Matrix GMs	10.2	408	97.2	78
2023	Mixed-Matrix GMs	8.5	382.5	27.3	78
2023	Mixed-Matrix GMs	5.9	266.3	45.1	78
2023	Mixed-Matrix GMs	4.8	479	30	79
2023	Mixed-Matrix GMs	28.1	5608	39	80
2025	Mixed-Matrix GMs	7.2	725	40	81
2007	Nanofiber-based MMMs	6.2	155	30	38
2008	Nanofiber-based MMMs	216.5	866	4.1	39
2014	Nanofiber-based MMMs	9.3	743	108	82
2016	Nanofiber-based MMMs	8.1	567	70	40
2019	Nanofiber-based MMMs	43	110	45	83
2019	Nanofiber-based MMMs	171	128.2	122	43
2021	Nanofiber-based MMMs	6.9	343	65	42

2024	Nanofiber-based MMMs	2.7	202.9	83.9	41
2018	Nanosheet-based MMMs	900	270	45	84
2019	Nanosheet-based MMMs	40	28	77	35
2020	Nanosheet-based MMMs	1986	139	41.4	32
2022	Nanosheet-based MMMs	521	2084	40	31
2023	Nanosheet-based MMMs	32.1	641	70	34
2024	Nanosheet-based MMMs	11.7	1264	64.3	33
2024	Nanosheet-based MMMs	4.6	460	63	36
2024	Nanosheet-based MMMs	6.6	462	47.5	37
2025	Nanosheet-based MMMs	23.8	40.5	36.2	85
2015	Zeolite-based MMMs	34.6	1245	24	24
2022	Zeolite-based MMMs	/	8300	60	86
2023	Zeolite-based MMMs	2.2	144	18	26
2024	Zeolite-based MMMs	230.7	6921	17.7	25
2021	POP-based MMMs	4.5	540	61.3	27
2021	POP-based MMMs	1.9	394	22.7	28
2021	POP-based MMMs	3.2	128.7	20.9	28
2021	POP-based MMMs	1.8	73.5	22.5	28
2021	POP-based MMMs	5	200.4	19.8	28
2021	POP-based MMMs	9.8	142	19.1	28
2021	POP-based	0.4	52	24.8	29

	MMMs				
2021	POP-based MMMs	25.9	1036	24.1	30
2025	POP-based MMMs	4.3	342.3	33.4	87
2018	COF-based MMMs	12.5	1200	33	19
2019	COF-based MMMs	157.2	7862	23.9	22
2024	COF-based MMMs	457	9137.7	20.2	20
2024	COF-based MMMs	15.6	1408	37	21
2024	COF-based MMMs	64.4	6440	17.7	23
2012	MOF-based MMMs	291	145	97	16
2013	MOF-based MMMs	8.5	250	23	4
2016	MOF-based MMMs	14.7	660	26.5	3
2016	MOF-based MMMs	24.1	482	41	15
2017	MOF-based MMMs	92.4	2000	24	2
2018	MOF-based MMMs	24	15	40	5
2019	MOF-based MMMs	488	132	31.9	6
2019	MOF-based MMMs	1295	1000	91	7
2020	MOF-based MMMs	10.6	400	25	9
2021	MOF-based MMMs	39	97.5	31.3	8
2021	MOF-based MMMs	152	304	78	13
2022	MOF-based MMMs	8.9	306	79	10
2022	MOF-based MMMs	301	300	91	12

2022	MOF-based MMMs	8	200	68	14
2022	MOF-based MMMs	/	500	25	88
2023	MOF-based MMMs	/	2841	36	89
2023	MOF-based MMMs	3577.4	9659	21.5	90
2024	MOF-based MMMs	1017	610.2	33	91
2024	MOF-based MMMs	2.1	210	130	92
2024	MOF-based MMMs	1.2	111.5	92.2	18
**2025**	**pure GMs**	**12.7**	**52.1**	**74**	**Our work**
**2025**	**NIGMs**	**211.0**	**844.0**	**151**	**Our work**

Supplementary Table 3 Comparison of the NIGMs and reported GMs and MMMs in terms of the CO_2/CH_4 separation performances.

Year	Membrane type	CO_2 permeance (GPU)	CO_2 permeability (Barrer)	CO_2/CH_4 selectivity	References
2017	pure GMs	4.7	190	24.4	50
2018	pure GMs	1.25	100	22	53
2020	pure GMs	0.2	20.4	118.6	54
2020	pure GMs	2.9	292	14.5	55
2021	pure GMs	1.3	66.9	11.4	56
2022	pure GMs	3.5	319.1	16.1	57
2022	pure GMs	6.5	456.4	18.1	60
2024	pure GMs	2.0	186.4	24.6	62
2016	Mixed-Matrix GMs	4.8	240	35	93
2016	Mixed-Matrix GMs	5.2	260	90	93
2016	Mixed-Matrix GMs	13.6	680	38	93
2019	Mixed-Matrix GMs	0.4	47	42	94
2021	Mixed-Matrix GMs	0.8	98	7	75
2022	Mixed-Matrix GMs	0.7	90.6	10.5	77
2023	Mixed-Matrix GMs	4.8	479	17	79
2025	Mixed-Matrix GMs	7.2	725	22	81
2016	Nanofiber-based MMMs	8.1	567	35	40
2020	Nanosheet-based MMMs	1986	139	15	32
2022	Nanosheet-based MMMs	521	2084	40.4	31
2025	Nanosheet-based MMMs	23.8	40.4	30	85
2015	Zeolite-based MMMs	34.6	1245	31	24
2022	Zeolite-based MMMs	/	8300	423	86

2021	POP-based MMMs	1.9	394	18.9	28
2021	POP-based MMMs	3.2	128.7	21.0	28
2021	POP-based MMMs	1.8	73.5	26.5	28
2021	POP-based MMMs	5	200.4	10.4	28
2021	POP-based MMMs	9.8	142	19.5	28
2021	POP-based MMMs	0.4	52	19.3	29
2021	POP-based MMMs	25.9	1036	28	30
2018	COF-based MMMs	12.5	1200	30	19
2024	COF-based MMMs	15.6	1408	39	21
2024	COF-based MMMs	64.4	6440	13.4	23
2012	MOF-based MMMs	291	145	30	16
2016	MOF-based MMMs	14.7	660	28	3
2019	MOF-based MMMs	1295	1000	38	7
2022	MOF-based MMMs	6.2	620	40	95
2022	MOF-based MMMs	1.1	11.5	39.5	17
2022	MOF-based MMMs	/	500	40	88
2023	MOF-based MMMs	3577.4	9659	18	90
2024	MOF-based MMMs	1017	610.2	13	91
2024	MOF-based MMMs	5.1	558	37.5	11
2025	MOF-based MMMs	5.1	851.3	25.9	96
**2025**	**pure GMs**	**12.7**	**52.1**	**22**	**Our work**

**2025**	**NIGMs**	**211.0**	**844.0**	**47**	**Our work**

Supplementary References

1. G. S. Larsen *et al., Macromolecules* **44**, 6944–6951 (2011).
2. M. Etxeberria-Benavides *et al., J. Membr. Sci.* **550**, 198–207 (2018).
3. A. Sabetghadam *et al., Adv. Funct. Mater.* **26**, 3154–3163 (2016).
4. T.-H. Bae, J. R. Long*, Energy Environ. Sci.* **6**, 3565–3569 (2013).
5. N. Prasetya, B. C. Donose, B. P. Ladewig*, J. Mater. Chem. A* **6**, 16390–16402 (2018).
6. N. U. Kim, B. J. Park, J. H. Lee, J. H. Kim*, J. Mater. Chem. A* 7, 14723–14731 (2019).
7. R. Xu *et al., J. Membr. Sci.* **573**, 455–464 (2019).
8. J. Wang *et al.*, *ACS Appl. Mater. Interfaces* **13**, 50441–50450 (2021).
9. J. Deng, Z. Dai, L. Deng*, Ind. Eng. Chem. Res.* **59**, 14458–14466 (2020).
10. R. Ding *et al., Sep. Purif. Technol.* **289**, 120768 (2022).
11. J. Wang, L. Li, J. Zhang, X. Li, *J. Membr. Sci.* **697**, 122569 (2024).
12. X. Zhang, X. Ren, Y. Wang, J. Li*, Sep. Purif. Technol.* **303**, 122195 (2022).
13. Y. Wang *et al., Sep. Purif. Technol.* **270**, 118800 (2021).
14. W. Zheng *et al., J. Membr. Sci.* **650**, 120330 (2022).
15. H. Zhu *et al., ACS Appl. Mater. Interfaces* **8**, 22696–22704 (2016).
16. T. Li, Y. Pan, K.-V. Peinemann, Z. Lai*, J. Membr. Sci.* **425–426**, 235–242 (2013).
17. E. Li *et al.*, *Sep. Purif. Technol.* **289**, 120714 (2022).
18. W. Zhao *et al., J. Appl. Polym. Sci.* **141**, e56009 (2024).
19. Y. Cheng *et al., J. Mater. Chem. A* **7**, 4549–4560 (2019).
20. Q. Chang *et al., Sep. Purif. Technol.* **330**, 125518 (2024).
21. S. Mashhadikhan *et al., J. Environ. Chem. Eng.* **12**, 113965 (2024).
22. G. Yu *et al., J. Membr. Sci.* **591**, 117343 (2019).
23. A. Riaz *et al., Sep. Purif. Technol.* **343**, 127175 (2024).
24. B. Zornoza *et al., AIChE J.* **61**, 4481–4490 (2015).
25. S. Han *et al.*, *ACS Appl. Polym. Mater.* **6**, 8640–8650 (2024).
26. A. Sohail, M. Sarfraz, S. Nawaz, Z. Tahir*, J. Cleaner Prod.* **399**, 136617 (2023).
27. R. Li *et al.*, *J. Membr. Sci.* **690**, 122203 (2024).
28. C. Soto *et al., Polymers* **13**, 931 (2021).
29. L. Rodríguez-Jardón, M. López-González, M. Iglesias, E. M. Maya, *J. Membr. Sci.* **619**, 118795 (2021).
30. C. Aguilar-Lugo *et al., ACS Appl. Polym. Mater.* **3**, 5224–5235 (2021).
31. W. Luo, Z. Niu, P. Mu, J. Li, *Macromolecules* **55**, 9851–9859 (2022).
32. A. A. Shamsabadi *et al., ACS Appl. Mater. Interfaces* **12**, 3984–3992 (2020).
33. C. Wang *et al., ACS Appl. Mater. Interfaces* **16**, 14152–14161 (2024).
34. M. Zhao *et al., Sep. Purif. Technol.* **324**, 124512 (2023).
35. A. Sabetghadam *et al., J. Membr. Sci.* **570–571**, 226–235 (2019).
36. P.-C. Wu, H.-Y. Wang, D.-Y. Kang, K.-L. Tung*, J. Membr. Sci.* **702**, 122797 (2024).
37. Q.-Q. Sun *et al., J. Appl. Polym. Sci.* **141**, e55906 (2024).
38. H. Cong, J. Zhang, M. Radosz, Y. Shen*, J. Membr. Sci.* **294**, 178–185 (2007).
39. H.-H. Tseng *et al., Desalination* **240**, 40–45 (2009).
40. H. Zhang, R. Guo, J. Hou, Z. Wei, X. Li*, ACS Appl. Mater. Interfaces* **8**, 29044–29051 (2016).
41. W. Zheng *et al., J. Membr. Sci.* **697**, 122500 (2024).
42. T. Sun *et al., J. Membr. Sci.* **639**, 119749 (2021).
43. Y. Wang *et al., J. Membr. Sci.* **589**, 117246 (2019).

44. P. Li, D. R. Paul, T.-S. Chung, *Green Chem.* **12**, 1052-1063 (2012).
45. P. T. Nguyen *et al., Ind. Eng. Chem. Res.* **52**, 8812 (2013).
46. J. Zhou *et al., Ind. Eng. Chem. Res.* **53**, 20064 (2014).
47. F. Moghadam, E. Kamio, A. Yoshizumia, H. Matsuyama*, Chem. Commun.* **51**, 13658–13661 (2015).
48. W. M. McDanel *et al., J. Membr. Sci.* **492**, 303 (2015).
49. X. Jiang, S. Li, L. Shao*, Energy Environ. Sci.* **10**, 1339 (2017).
50. E. G. Estahbanati, M. Omidkhah, A. E. Amooghin, *J. Ind. Eng. Chem.* **51**, 77–89 (2017).
51. F. Moghadam *et al., J. Membr. Sci.* **530**, 166 (2017).
52. M. Longo *et al., Polymer* **156**, 22–29 (2018).
53. V. A. Kusuma *et al., J. Membr. Sci.* **545**, 292 (2018).
54. I. Kammakakam *et al., ACS Sustainable Chem. Eng.* **8**, 5954 (2020).
55. P. Bernardo, D. Zampino, G. Clarizia*, Sep. Purif. Technol.* **250**, 117201 (2020).
56. M. Klepić *et al., Sep. Purif. Technol.* **270**, 118812 (2021).
57. Y. Fu *et al., J. Mater. Chem. A* **10**, 4695–4702 (2022).
58. J. Zhang *et al., Ind. Eng. Chem. Res.* **61**, 4648 (2022).
59. J. Zhang *et al., J. Membr. Sci.* **663**, 121032 (2022).
60. H. J. Min *et al., J. Membr. Sci.* **660**, 120837 (2022).
61. S. He *et al., J. Membr. Sci.* **685**, 121912 (2023).
62. J. Xiao *et al., Angew. Chem. Int. Ed.* **63**, e202411270 (2024).
63. S. He *et al., J. Membr. Sci.* **695**, 122482 (2024).
64. S. He *et al., J. Membr. Sci.* **711**, 123200 (2024).
65. Y. Yu *et al., Sep. Purif. Technol.* **331**, 125591 (2024).
66. J. Zhang *et al., Sep. Purif. Technol.* **359**, 130499 (2025).
67. Y. Yu *et al., Sep. Purif. Technol.* **362**, 131916 (2025).
68. F. Ranjbaran, E. Kamio, H. Matsuyama, *J. Membr. Sci.* **544**, 252 (2017).
69. H. R. Mahdavi *et al., J. Nat. Gas Sci. Eng.* **46**, 275 (2017).
70. F. Ranjbaran, E. Kamio, H. Matsuyama, *Ind. Eng. Chem. Res.* **56**, 12763–12772 (2017).
71. W. Fam *et al., Ind. Eng. Chem. Res.* **58**, 3304 (2019).
72. W. G. Lee, S. W. Kang*, Chem. Eng. J.* **356**, 312–317 (2019).
73. J. Zhang *et al., Ind. Eng. Chem. Res.* **60**, 12640 (2021).
74. J. Zhang *et al., Ind. Eng. Chem. Res.* **60**, 12698–12708 (2021).
75. A. R. Nabais *et al., Membranes* **11**, 998 (2021).
76. E. Kamio *et al., Polym. J.* **53**, 137 (2021).
77. A. R. Nabais *et al. J. Membr. Sci.* **660**, 120841 (2022).
78. Y. Sun *et al. J. Membr. Sci.* **683**, 121818 (2023).
79. A. R. Nabais *et al. J. Membr. Sci.* **685**, 121938 (2023).
80. B. Zhu *et al., Angew. Chem. Int. Ed.* **63**, e202315607 (2024).
81. D. A. Sousa *et al., ACS Appl. Polym. Mater.* **7**, 5944 (2025).
82. S. Wang *et al., J. Membr. Sci.* **460**, 62 (2014).
83. R. Borgohain *et al., React. Funct. Polym.* **143**, 104331 (2019).
84. G. Huang *et al., J. Membr. Sci.* **565**, 370 (2018).
85. S. V. Prasad, G. Arthanareeswaran, *ACS Appl. Polym. Mater.* **7**, 6042 (2025).
86. X. Tan *et al., Science* 3**78**, 1189 (2022).
87. Y. Liu *et al., ACS Appl. Polym. Mater.* **7**, 4535 (2025).
88. S. J. Datta *et al., Science* 3**76**, 1080 (2022).

89. S. Li *et al., Angew. Chem. Int. Ed.* 63, e202315167 (2024).
90. T. H. Lee *et al., Nat Commun.* 14, 8330 (2023).
91. M. Kang *et al., J. Membr. Sci.* **698**, 122611 (2024).
92. Z. Yu *et al., ACS Appl. Polym. Mater.* **6**, 5443 (2024).
93. Z. V. Singh *et al., J. Membr. Sci.* **509**, 149 (2016).
94. C. A. Dunn *et al., Ind. Eng. Chem. Res.* **58**, 4704 (2019).
95. X. Lv *et al., ACS Appl. Mater. Interfaces* **14**, 49233 (2022).
96. L. Li *et al., J. Membr. Sci.* **720**, 123775 (2025).
