# Supplementary material for: Nanofiber-interwoven gel membranes with tunable 3D-interconnected transport channels for efficient CO2 separation
Source: Nat Commun. 2025 Sep 2;16:8199. doi: 10.1038/s41467-025-63502-2 (PMC12405441; doi:10.1038/s41467-025-63502-2)
Supplement: Supplementary file 2 — Description of Additional Supplementary Files [file 41467_2025_63502_MOESM2_ESM.pdf]

Title: Supplementary Movie 1

Description: The NIGMs consisting of 16 CNT molecules and 50 PEG chains were added into a cubic simulation box. Then, CO_2 molecules parameterized by the GAFF force field with the RESP charges were randomly packed into composite membrane system. During a 100 ns equilibrium NPT simulation, mean square displacements of CO_2 near PEG matrix and near CNT molecules were counted to eventually obtain their diffusion coefficients. The diffusion coefficients of CO_2 near the PEG matrix and near the CNT within the NIGMs were shown in Supplementary Fig. 18.
